# Evaluation of the Efficacy of BCG in Protecting Against Contact Challenge With Bovine Tuberculosis in Holstein-Friesian and Zebu Crossbred Calves in Ethiopia

**DOI:** 10.3389/fvets.2021.702402

**Published:** 2021-07-22

**Authors:** Berecha Bayissa, Asegedech Sirak, Adane Worku, Aboma Zewude, Yemisrach Zeleke, Mahlet Chanyalew, Balako Gumi, Stefan Berg, Andrew Conlan, R. Glyn Hewinson, The ETHICOBOTS Consortium, James L. N. Wood, H. Martin Vordermeier, Gobena Ameni

**Affiliations:** ^1^Animal Health and Zoonotic Research Unit, Aklilu Lemma Institute of Pathobiology, Addis Ababa University, Addis Ababa, Ethiopia; ^2^Vaccine Production and Drug Formulation Directorate, National Veterinary Institute, Debre Zeit, Ethiopia; ^3^National Animal Health Diagnostic and Investigation Centre, Sebeta, Ethiopia; ^4^Animal and Plant Health Agency, Addlestone, United Kingdom; ^5^Disease Dynamics Unit, Department of Veterinary Medicine, University of Cambridge, Cambridge, United Kingdom; ^6^Institute of Biology, Environmental and Rural Sciences, Aberystwyth University, Ceredigion, United Kingdom; ^7^Department of Veterinary Medicine, College of Food and Agriculture, United Arab Emirates University, Al Ain, United Arab Emirates

**Keywords:** bovine tuberculosis, crossbred cattle, efficacy of BCG, natural transmission, Ethiopia

## Abstract

Bovine tuberculosis (bTB) is prevalent in intensive dairy farms in Ethiopia. Vaccination could be an alternative control approach given the socio-economic challenges of a test-and-slaughter control strategy. The efficacy of the BCG was evaluated on 40 Holstein-Friesian (HF) and zebu crossbred calves recruited from single intradermal cervical comparative tuberculin (SICCT) test negative herds and randomly allocated into two groups. Twenty-two calves were vaccinated within 2 weeks of age, and 18 were kept as a control. Six weeks post-vaccination, the two groups were exposed and kept mixed with known SICCT test positive cows for 1 year. Immune responses were monitored by interferon gamma (IFN-γ) release assay (IGRA), SICCT test, and antibody assay. Vaccinated calves developed strong responses to the SICCT test at the sixth week post-vaccination, but did not respond to ESAT-6/CFP-10 peptide antigen-based IGRA. During the exposure, IFN-γ response to the specific peptide cocktail [*F*_(2.44, 92.67)_ = 26.96; *p* < 0.001] and skin reaction to the specific proteins cocktail [*F*_(1.7, 64.3)_; *p* < 0.001] increased progressively in both groups while their antibody responses were low. The prevalence of bTB was 88.9% (95% CI: 65.3–98.6) and 63.6% (95% CI: 40.7–83.8) in the control and vaccinated calves, respectively, based on *Mycobacterium bovis* isolation, giving a direct protective efficacy estimate of 28.4% (95% CI: −2.7 to 50.1). The proportion of vaccinated calves with lesion was 7.0% (34/484) against 11.4% (45/396) in control calves, representing a 38% (95% CI: 5.8–59.4) reduction of lesion prevalence. Besides, the severity of pathology was significantly lower (Mann–Whitney *U*-test, *p* < 0.05) in vaccinated (median score = 2.0, IQR = 0–4.75) than in control (median score = 5, IQR = 3.0–6.25) calves. Moreover, survival from *M. bovis* infection in vaccinated calves was significantly (log-rank test: χ^2^ = 6.749, *p* < 0.01) higher than that of the control calves. In conclusion, the efficacy of BCG was low, but the reduced frequency and severity of lesion in vaccinated calves could suggest its potential role in containing onward transmission.

## Introduction

In Ethiopia, bTB is an endemic cattle disease (1) characterized by the progressive development of granulomatous lesions in the respiratory tract and other tissues of the infected host (2). The disease in Ethiopia is mainly caused by *Mycobacterium bovis*, but may be associated with other members of the *Mycobacterium tuberculosis* complex in Ethiopia and other countries (3, 4). The prevalence of bTB in Ethiopia varies with the type of production system and breed of cattle (5–9). Intensively reared herds, consisting of pure Holstein-Friesian (HF) cows and/or their crosses with indigenous zebu breeds, were more commonly affected (10–13). The number of intensive dairy farms is increasing around urban centers in response to the prevailing demand for dairy products (11, 12). In addition to reducing production in infected cows (14), the disease can also be transmitted to humans, especially as consumption of raw milk and meat are both common in the Ethiopian society (10, 15). The animal and public health risks dictate that controls warrant implementation. The lack of funding to underpin a test and slaughter control program in Ethiopia, in common with other low- and middle-income countries (16), means that vaccination is an attractive alternative where the disease is endemic at high prevalence (17–19).

BCG could be a potential vaccine candidate for the control of tuberculosis in cattle (19–21). Several evaluations have been undertaken to determine a protective capacity of BCG in cattle since 1911 (20–23). The results of experimental trials are encouraging although different field trials demonstrated variable protective efficacies [reviewed by (21)]. Several explanations were given for the observed variability of efficacy of BCG including variation in the strain and dose of BCG used [reviewed by (21)], challenge model (5, 24), previous exposure to environmental mycobacteria (25, 26), and breed and health status of study animals (11, 27–29). In general, a recent meta-analysis reported a relatively low direct protection of BCG against infection with a pooled efficacy estimate of 25%, and significant reduction in the frequency and severity of pathology of bTB in vaccinated animals (19).

Two contact challenge efficacy trials were conducted in Ethiopia on pure HF breed neonatal calves in which efficacies varied between 56% (5) and 23% (12). Further studies are needed to resolve the large difference in reported efficacy of the vaccine and to consider its use in crossbred cattle (HF and zebu cross) preferred in Ethiopia for the expansion of the dairy sector, as no BCG efficacy trial has been conducted in them. This study assessed the capacity of the BCG vaccine to protect HF–zebu cross neonatal calves against *M. bovis* infection under natural transmission settings by housing vaccinated and non-vaccinated calves in a barn for 1 year together with cattle known to be tuberculin reactors. We also perform a pilot study of a protein cocktail with DIVA (Differentiating Infected from Vaccinated Animal) properties (30) to understand its potential as a skin test in distinguishing BCG vaccination from bovine TB infection in crossbred cattle.

## Materials and Methods

### Study Setting

The experiment was undertaken at the National Animal Health Diagnostic and Investigation Center (NAHDIC) at Sebeta, which is located 25 km southwest of Addis Ababa, the capital city of Ethiopia. Ethiopia is endemic to bTB and there is no national control strategy for bTB in place in the country so far. Thus, taking into account the endemicity of the diseases in the country, the local biosecurity and safety procedures were followed. Accordingly, an isolated barn with the holding capacity of 120 cattle was built in the compound of NAHDIC and used for this experiment. The barn was fenced with wire and completely isolated from the rest of the compound. The experimental animals were watered and fed inside this barn. The feces and urine were removed to a septic tank through pipes connected to the barn. The septic tank was built within the fenced area outside the barn. The animal attendants were educated on the risk of zoonosis and were obliged to use personal protective equipment all the time while working in the barn. After the completion of the experiment, all calves were slaughtered in the autopsy facilities of NAHDIC and the carcasses were disposed by incineration.

### Study Animals

A total of 60 single intradermal cervical comparative tuberculin (SICCT) test–positive cows with an average age of 4 years were purchased from Holeta Agricultural Research Center and used as sources of infection (seeders). A total of 82 naïve HF × zebu crossbred male neonatal calves were recruited at an average age of 1 week and served for the conducting of the experiment. The neonates were purchased from 57 SICCT test–negative dairy farms and screened for bTB by IGRA. Thereafter, they were randomly allocated into vaccinated and control groups. About half of the calves were vaccinated within 2 weeks of age while the other half were left to serve as controls. Both groups of calves were exposed to SICCT test–positive cows at 2 months of age and kept in contact with the cows for 1 year. The ratio of calf to seeder was 1:1 at the start of the exposure as in previous similar studies done in Ethiopia (5, 12). However, the ratio was reduced to 2:1 for the last 6 months of exposure due to reduced space within the barn from growth of the experimental calves. The calves were fed on pasteurized partially skimmed milk, grass hay, and concentrate up to the age of 4 months. Weaned calves and the reactor cows were fed on hay and concentrate. Water was supplied to both calves and cows *ad libitum*. The study was approved by the Ethical Review Board of the Aklilu Lemma Institute of Pathobiology, Addis Ababa University (IRB/07/2014). Unfortunately, about half of the calves could not complete the experiment because of mortality due to physical attack by cows, non-TB-related infections, and poor qualities of concentrate feeds and pasteurized milk. Thus, the analysis of the results of the study was conducted on the 22 vaccinated and 18 control calves that survived to the end of the study.

### Vaccination of Calves

The vaccine used in this experiment was a freeze-dried Russian strain BCG vaccine produced by BB-NCIPD Ltd., Sofia, Bulgaria, and distributed by InterVax Ltd., Toronto, Canada. Each calf in the vaccinated group was subcutaneously administered on the neck with 1 × 10^6^ CFU dose of BCG. The calves were vaccinated within 2 weeks of their birth to reduce the possible influence of environmental mycobacteria infection on the BCG efficacy (25, 26).

### SICCT Test

The SICCT test was conducted at four time points during the experiment, at the 6th week after vaccination or before exposure to reactor cows (month 0), and at the 4th, 8th, and 12th months post-exposure. This test was performed using bovine and avian purified protein derivatives (PPD-B and PPD-A) obtained from Thermo-Fisher (Prionics, Lelystad, the Netherlands). Briefly, 0.1 ml PPD-A (2,500 IU/ml) was administered intradermally on the left side of the neck at a site tip of ear reached and the same volume of PPD-B (3,000 IU/ml) was injected at a site 12 cm distant from the PPD-A site in the shoulder direction. The skin thicknesses were measured just before injection and at 72 h post-injection by the same operator using the same digital caliper, and the results were presented as change in skin thickness (mm) between the two readings. The differences in the increase of skin thickness at the bovine and avian PPD injection sites were determined. An animal was considered to be positive when the increase in skin thickness at the bovine PPD site was >4 mm more than the increase in skin thickness at the site of the avian injection. If the differential increase between the two sites were equal to or < 1mm, or between 1 and 4mm, the animal was considered negative and inconclusive, respectively (31).

### DIVA Prototype Skin Test

A DIVA skin test was conducted on the experimental calves at the same time as the SICCT test. The DIVA reagent contained a cocktail of recombinant proteins of the ESAT-6, CFP-10, and Rv3615c antigens of *M. tuberculosis* and *M. bovis* (Lionex GmbH, Germany). When preparing the DIVA reagent, equal amounts of each freeze-dried protein were combined in a PBS solution containing 100 μg/ml of each protein (300 μg total protein/ml). The solution was stored at −80°C until needed. Each animal was injected intradermally with 0.1 ml DIVA solution (30 μg total protein per dose) into the middle of the right side of the neck. Skin thickness was measured before inoculation and at 72 h post-inoculation. The measurements were done by the same operator using the same digital caliper in every testing. Results are expressed as the difference in skin fold thickness (in millimeters) before administration of the antigens and 72 h post-administration. Skin reaction was considered positive if the increase in skin thickness at the injection site was ≥2mm (20, 32).

### IFN-γ Release Assay

The IGRA was performed on the sentinels every 3 weeks after the time of vaccination and every 2 months after the start of exposure to the reactor cows. About 5 ml of blood was withdrawn from the jugular vein of each calf into a sodium heparinized Vacutainer tube (BD Medica, Dublin, Ireland). The blood samples were transported to ALIPB laboratory within 8 h of sampling at ambient temperature and 250 μl of whole blood was dispensed into flat bottom 96-well culture plates and stimulated with mycobacterial antigens in duplicates. Each sample was stimulated with 25 μl avian PPD (10 μg/ml), 25 μl bovine PPD (10 μg/ml), and 25 μl ESAT-6/CFP-10 peptide cocktail (10 μg/ml). Pokeweed mitogen (PWM; 10 μg/ml) and RPMI (25 μl per well) was used as positive and negative controls, respectively. After incubation for 48 h at 37°C in a humidified atmosphere with 5% CO_2_, plasma supernatants were harvested and stored at −80°C until used for IGRA. The IFN-γ level in the plasma supernatants were measured by the BOVIGAM kit (Prionics, Switzerland). For comparative analysis of PPD-B vs. PPD-A responses, a positive result was defined as a value for the OD_450_ PPD-B minus the OD_450_ with PPD-A of ≥0.1 and a value for the OD_450_ with PPD-B minus the OD_450_ saline stimulation of ≥0.1. Similar criteria were used to define positive result when the OD_450_ value of whole blood supernatant stimulated with ESAT-6/CFP-10 cocktail peptides minus the OD_450_ value of saline stimulation (24).

### IDEXX *M. bovis* Antibody ELISA

About 3 ml of blood samples was collected in 5 ml BD Vacutainer tubes for serological examination of the experimental animals. The samples were centrifuged at 3,500 rpm for 15 min to harvest their sera that were stored at −20°C until processed. The sera were tested for presence of *M*. *bovis*–specific antibodies using the IDEXX *M. bovis* antibody ELISA (IDEXX Laboratories, USA). Serum samples and kit controls were diluted at 1:50 dilution and tested in duplicate. The test was performed according to the manufacturer's instruction. Criteria for the test validity were an OD_450_ ≥ 0.3 for the positive control and an OD_450_ ≤ 0.2 for the negative control. Results were presented as OD_450_ value sample-to-positive control ratio (S/P) calculated by subtracting the mean OD_450_ value of kit negative control from each sample and dividing this value by the corrected positive-control value (mean OD_450_ of positive control minus mean OD_450_ of negative control). Samples with S/P ratio of ≥0.30 were considered positive for *M. bovis* antibodies.

### Post-mortem and Histopathological Examinations

Experimental calves were humanely slaughtered and their carcasses were disposed by incineration. A detailed post-mortem inspection was performed by a pathologist without previous knowledge of their vaccination status. Lymph nodes (parotid, mandibular, retropharyngeal, mediastinal, bronchial, tracheobronchial, hepatic, and mesenteric) and seven lobes of lung were examined in detail. The severity of lesions was determined by applying the semi-quantitative procedure according to Vordermeier et al. (33). Both lymph node and lung pathology scores were summed up to a total pathology score for each animal. Suspicious lesions were collected for both histopathological and bacterial examinations. Tissues for histopathological examination were fixed in 10% buffered formalin. The fixed tissue specimens were processed using SHANDON Citadel 1000 tissue processor, dehydrated using graded ethyl alcohol, and then the alcohol was cleared using xylene. Then, the tissue was impregnated in melted paraffin using TEC2900 modular Tissue Embedding Centre (Histo-Line Laboratories). The embedded tissue specimens were trimmed to 4 μm thickness and the tissue sections were stained with H&E and Ziehl–Neelsen (ZN) for detection of TB granuloma and acid-fast bacilli (AFB), respectively. Slides were examined by light microscopy to determine the distribution of granuloma development stages as defined by Wangoo et al. (34). Four stages of granulomas were quantified and analyzed as previously described (35).

### Isolation of Mycobacteria

Collection of tissue specimens and isolation of mycobacteria from those tissues were performed following the OIE manual (31). At post-mortem, tissue specimens from lymph nodes and lungs were collected into sterile universal bottles in 5 ml of a 0.9% saline solution and transported to the laboratory for bacterial isolation. For calves with visible lesion, only lesioned tissues were processed for culture, while for calves with non-visible lesion, all head, thoracic, and abdominal lymph nodes were processed. For bacteria cultures, the tissue samples were homogenized with pestle and mortar, decontaminated by adding an equal volume of 4% NaOH, centrifuged at 3,000 rpm for 15 min, and neutralized with 1% (0.1 N) HCI with phenol red as an indicator. Finally, the suspensions were inoculated on two slants of Löwenstein–Jensen media, one enriched with sodium pyruvate and one with glycerol, and incubated at 37°C for up to 8 weeks. Slants were monitored on a weekly basis. Slants with no growth at week 8 were considered negative. The colonies on the slants were harvested, heat killed at 85°C for 1 h in a water bath for the release of DNA from the isolates, and then used for mycobacterial species identification using region of difference (RD) 4-based polymerase chain reaction (PCR) following the procedure used by Firdessa et al. (10). Spoligotyping was performed for identification of spoligotypes of the isolates according to Kamerbeek et al. (36), using a non-commercial biodyne-C-membrane produced by the Animal and Plant Health Agency (UK).

### Data Management and Statistical Analysis

Statistical analysis and graphs were made using GraphPad Prism 8. A descriptive analysis was summarized using median and interquartile range (IQR). Tuberculosis status of the study calves was defined by the detection of typical TB lesions in the tissues, by culture positivity for *M. bovis*, or by both criteria. Comparisons of TB gross pathology and culture positivity between vaccinated and non-vaccinated animals were made using Fisher's exact-test. Direct vaccine efficacy (protection from infection) was estimated with the formula described by Orenstein et al. (37), which considers the incidence rates of disease in the vaccinated and unvaccinated calves; i.e., vaccine efficacy is the percentage reduction in the incidence rate of disease among vaccinated calves compared with the unvaccinated calves. Chi-square and Fisher's exact tests were used to compare the percentages of macroscopic and microscopic pathology in different tissues of vaccinated and control calves. The degree of gross pathology and bacterial load within affected tissues between BCG-vaccinated and control calves were compared using the Mann–Whitney *U*-test. The specific IFN-γ and antibody response concentrations (optical density values at 450 nm, OD_450_) and skin thickness change over sampling period in each group were compared using Friedman test (repeated measures non-parametric analysis of variance) with Dunn's multiple comparison test, and repeated measures analysis of variance with Geisser–Greenhouse correction for comparing between the groups. The differences in immunological responses (DIVA skin test, DIVA IGRA, and IDEXX ELISA) between control and vaccinated groups at each testing point were analyzed using the Bonferroni multiple comparison test. A log-rank test (survival analysis) was used to compare the incidence rate of *M. bovis* infection in the control and vaccinated groups. The non-parametric Spearman correlation was used to assess correlation between antibody response and pathological score as well as between IFN-γ response to ESAT-6/CFP-10 peptide cocktail and pathological score at the end of the experiment. A χ^2^-test for trend was performed to compare the distribution of stage I–IV lesions in vaccinated and control groups. In all cases, a 95% confidence level and a significance level of 5% were used to assess statistical significance.

## Results

### Post-vaccination and Pre-exposure Cellular and Humoral Immune Responses

#### Cellular Immune Response

**Figure 1:**
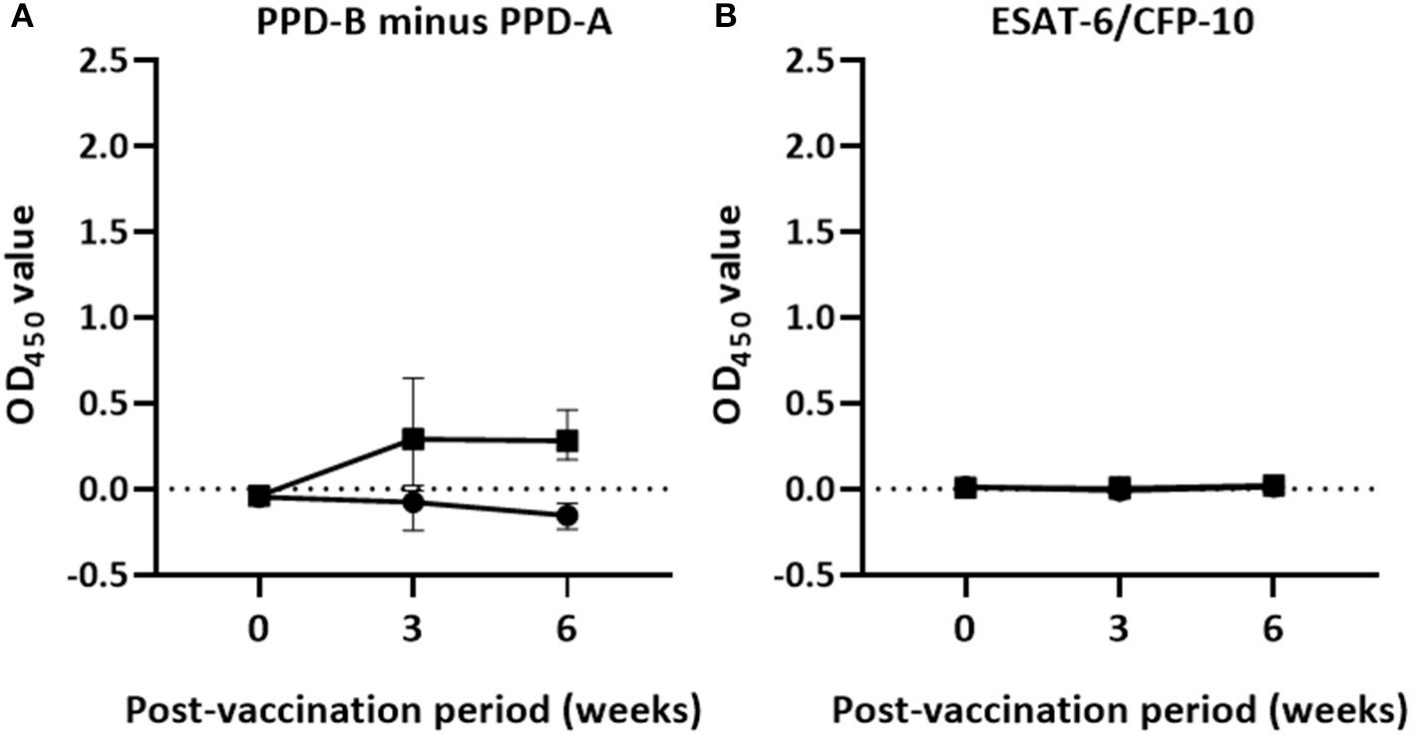
Post-vaccination IFN-γ responses in the experimental calves. The figure describes Antigen-specific IFN-γ production in whole blood cultures of 22 vaccinated and 18 control calves 6 weeks after vaccination. The released IFN-γ was measured from whole blood stimulated with PPDs **(A)** or specific peptides cocktail **(B)** using the BOVIGAM assay. There was a statistically significant difference (*p* < 0.0001) in IFN-γ responses to PPD (B-A) in vaccinated and control calves at the 3rd and the 6th week while the IFN-γ response to the peptides cocktail was not detectable in both groups of calves after vaccination. Error bars indicate the median (±95% CI) OD_450_ value of the two groups for each time point.

The dynamics of IFN-γ responses in the peripheral circulation of vaccinated and non-vaccinated calves in response to mycobacterial antigens is presented in Figure 1. IFN-γ responses to PPD (B-A) and ESAT-6/CFP-10 peptide cocktail–specific IFN-γ response were low in both groups of calves before vaccination, i.e., on day 0 (Figure 1). However, the IFN-γ response to PPD (B-A) increased after vaccination in the vaccinated calves attaining a peak at the third week post-vaccination with median OD_450_ value of 0.29 (IQR = 0.02–0.67) and remained higher with median OD_450_ value of 0.28 (IQR = 0.17–0.47) at the sixth week post-vaccination. At the third week vaccination, 59.1% of vaccinated calves were positive in the differential IFN-γ responses to PPD-B minus PPD-A while all of them were positive at the sixth week post-vaccination. The IFN-γ responses to PPD (B-A) in vaccinated calves significantly (*p* < 0.0001) increased over post-vaccination. However, the PPD-B-specific IFN-γ response of non-vaccinated calves stayed lower and less than that of their PPD-A-specific IFN-γ response. Therefore, the differential IFN-γ response to PPD-B of the vaccinated calves was significantly (*p* < 0.0001) greater than that of the control calves at the third and sixth week post-vaccination (Figure 1A). However, the ESAT-6/CFP-10 peptide-specific IFN-γ response remained at a low level in both treatment groups during the post-vaccination period (Figure 1B).

**Figure 2:**
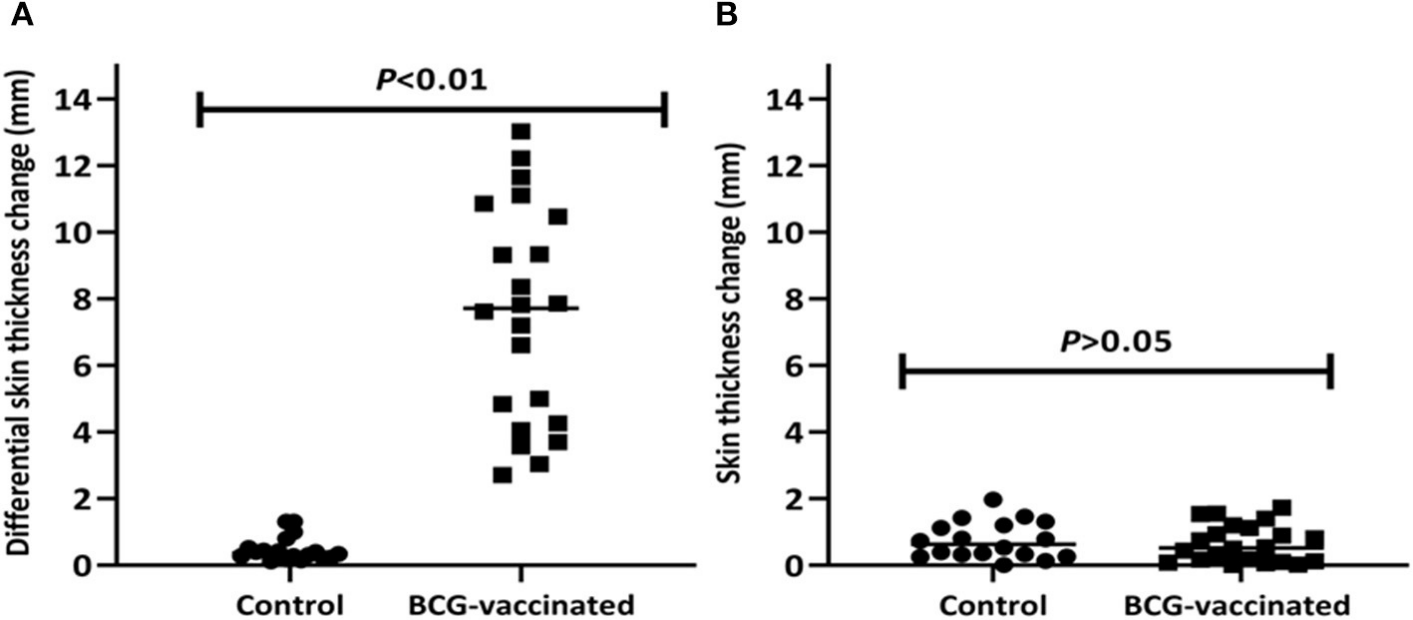
Post-vaccination skin test responses in the experimental calves. The figure shows skin reaction to the PPD (B-A) **(A)** and to the DIVA proteins cocktail **(B)** in individual vaccinated and control calves after vaccination. The individual animal result is represented by a solid circle for the control group and a solid square for the vaccinated group, and medians are indicated by horizontal lines. The change in skin thickness due to the PPD (B-A) was significantly (*p* < 0.01) higher in the vaccinated calves than in the control calves while the change to the DIVA proteins cocktail was not observed in both groups of calves.

The median difference of skin thickness at the injection sites of PPD-B and PPD-A was 7.7mm (IQR = 4.3–10.6) in BCG-vaccinated calves at the sixth week post-vaccination; the median difference in unvaccinated calves was 0.4mm (IQR = 0.2–0.6). Among these vaccinates, 82% (*n* = 18) were positive in the SICCT test and this also meant that the difference in SICCT response between the two groups were significantly (*p* < 0.001) different (Figure 2A). The DIVA protein cocktail induced <2mm skin thickness reaction in both vaccinated and control calves (Figure 2B).

#### Humoral Immune Response

**Figure 3:**
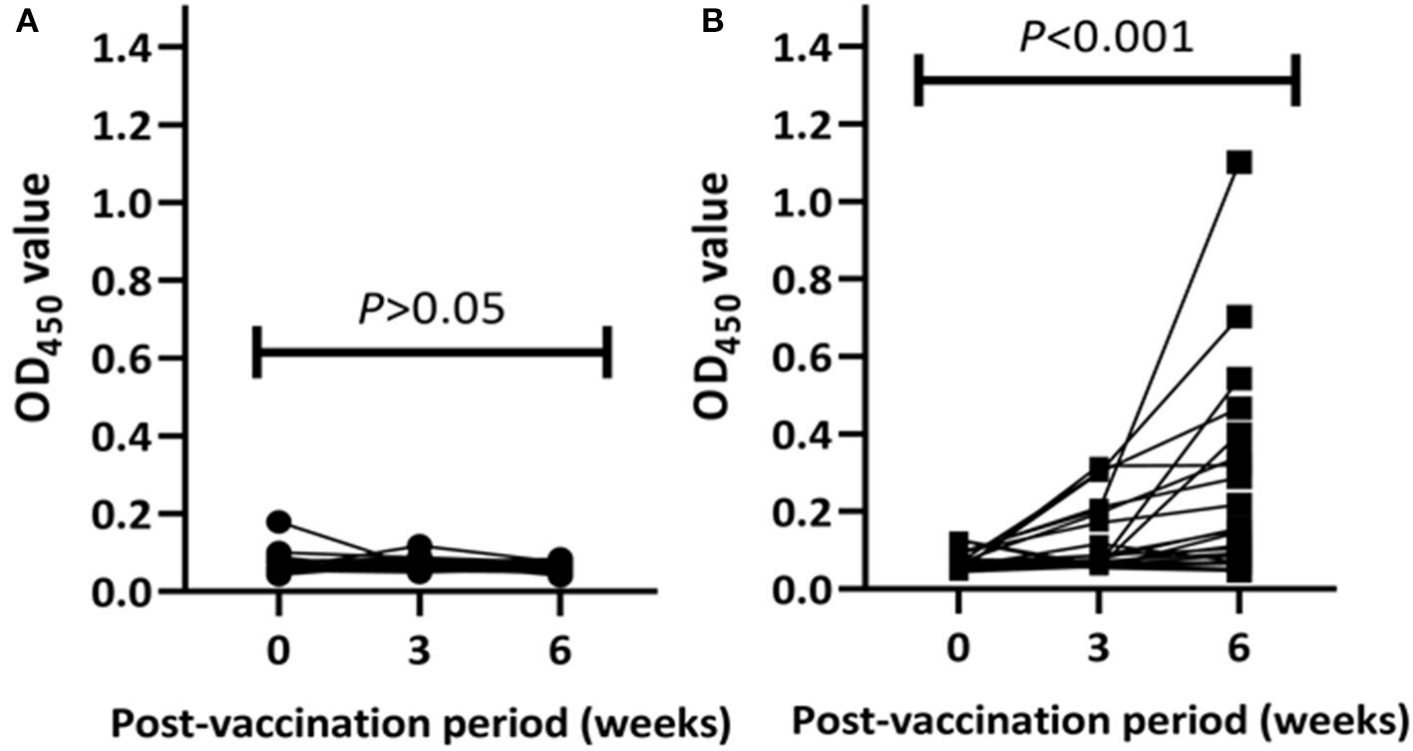
Post-vaccination antibody responses in the experimental calves. Kinetics *M. bovis* specific antibody responses in individual control **(A)** and vaccinated **(B)** calves are presented in the figure. The antibody response was expressed as optical density (OD) value at 450 nm and the values of each animal are connected with lines. The antibody response in the control calves remained at low levels throughout the post-vaccination period while the response in the vaccinated calves increased significantly at week 6 post-vaccination (Friedman test, *p* < 001).

Post-vaccination humoral immune responses were assessed using the IDEXX *M. bovis* ELISA. On the day of BCG vaccination, the OD_450_ value of the antibody response in all BCG-vaccinated and control calves was < 0.18. Antibody responses in unvaccinated calves were undetected over the post-vaccination period (Figure 3A). In vaccinated calves, the median OD_450_ value of specific antibody responses were 0.075 (IQR = 0.06–0.20) at the third week and increased to 0.15 (IQR = 0.07–0.36) at the sixth week (Figure 3B). Out of the 22 vaccinated calves, three and an additional five calves developed detectable *M. bovis* protein-specific antibody responses at the third and the sixth weeks of vaccination, respectively. In general, the antibody responses in vaccinated calves were significantly (*p* < 0.001) increased during the 6 weeks of post-vaccination period (Figure 3).

### Post-exposure Cellular and Humoral Immune Responses

#### Cellular Immune Response

**Figure 4:**
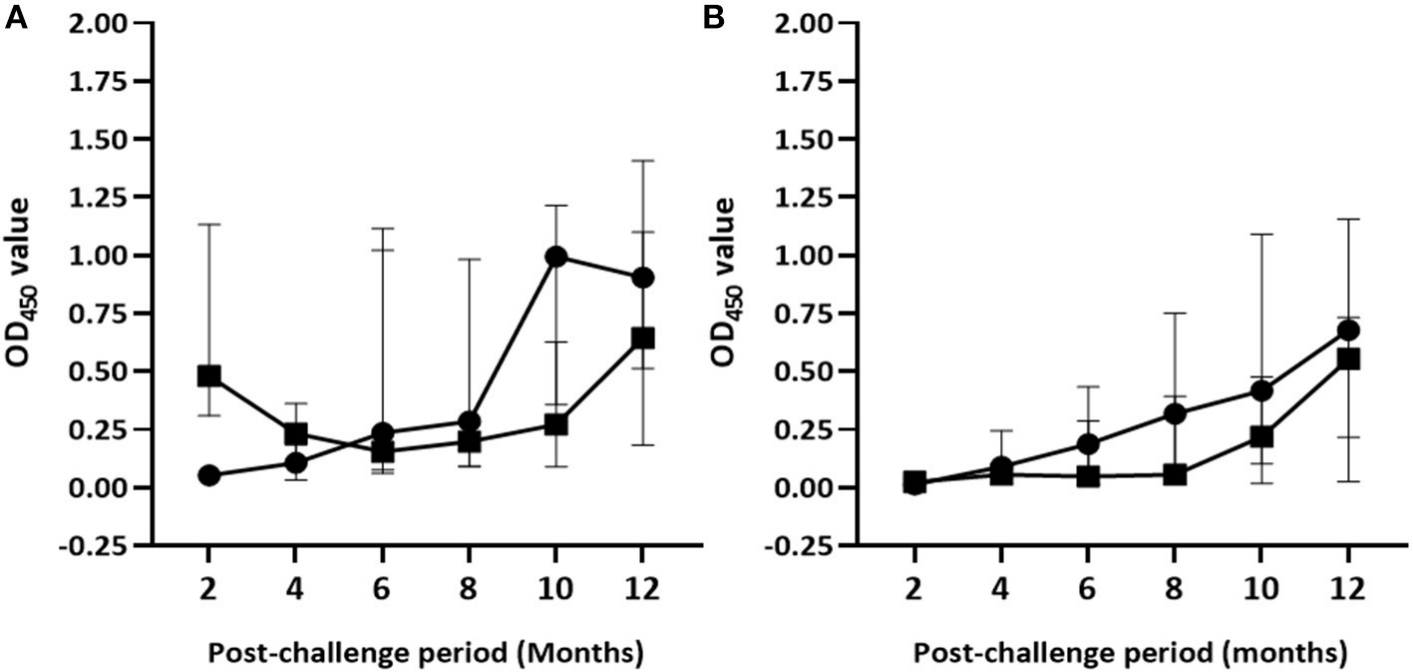
Post-exposure IFN-γ responses in the experimental calves. The figure demonstrates kinetics of IFN-γ response to PPD (B-A) **(A)** and ESAT-6/CFP-10 peptides cocktail **(B)** in the control and vaccinated calves. The IFN-γ response was measured by optical density units (OD_450_). Solid circle and solid square error bars indicate the median (±95% CI) OD_450_ value of control and vaccinated calves, respectively.

**Figure 5:**
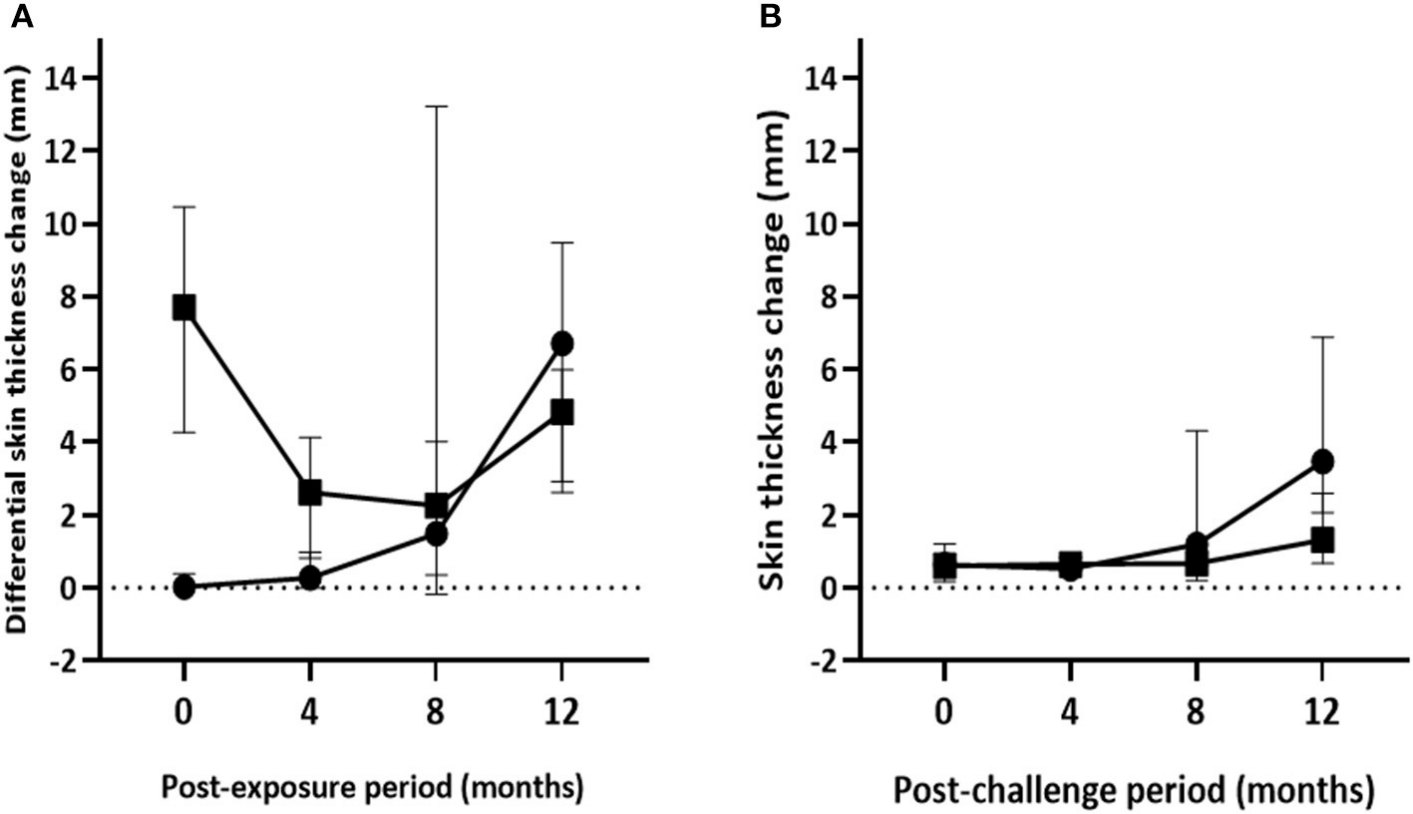
Post-exposure skin test responses in the experimental calves. The figure presents kinetics of skin reactions (in mm) to the PPD (B-A) **(A)** and the DIVA proteins cocktail **(B)** injections in the vaccinated and control calves during 12 months of exposure. Solid circle and solid square represent the median (±95%CI) in the control and vaccinated calves at each time point, respectively.

The median of IFN-γ response to PPD (B-A) and skin reaction to PPD (B-A) in control calves significantly increased throughout the exposure period. Median OD_450_ values of PPD (B-A)–based IFN-γ response progressively increased from −0.04 (IQR = −0.08 to 0.02) at the second month to 0.91 (IQR = 0.49–1.46) at the end of the experiment (Figure 4A). Likewise, median skin reaction to PPD (B-A) gradually rose up from 0.3mm (IQR = −0.03 to 2.36) at the 4th month to 7.9mm (IQR = 3.7–11.2) at the 12th month post-exposure in the control calves (Figure 5A). The number of calves that were positive in the SICCT test at the 4th, 8th, and 12th months were 4, 10, and 14, respectively. In contrast, the IFN-γ response to PPD (B-A) (Figure 4A) and skin reaction to PPD (B-A) (Figure 5A) in vaccinates declined during the first 6 months of exposure, and then progressively increased during the second half duration of the experiment. Median skin reaction to PPD (B-A) in vaccinated calves were 2.6mm (IQR = 0.8–4.2), 3.0mm (IQR = 0.3–5.4), and 4.8mm (IQR = 2.9–6.1) at the 4, 8, and 12th months of exposure.

On the other hand, the magnitude of ESAT-6/CFP-10 peptide cocktail–specific IFN-γ responses gradually increased in both vaccinated and control groups [*F*_(2.44, 92.67)_ = 26.96; *p* < 0.001] during the exposure although the intensity of the response was stronger [*F*_(1, 38)_ = 9.43; *p* < 0.01] in control calves than in the vaccinated calves (Figure 4B). However, *post-hoc* multiple comparison tests did not suggest the difference between vaccinated and non-vaccinated calves at each post-exposure testing point was statistically significant (Bonferroni test, *p* > 0.05). Furthermore, intensity of ESAT-10/CFP-10-based IFN-γ response showed a moderate correlation (*r* = 0.51, 95% CI = 0.22–0.71, *p* < 0.001) to pathology score.

The highest median skin reaction to DIVA proteins in BCG-vaccinated calves was 1.3mm (IQR = 0.6–2.6) at the end of the experiment with seven test-positive calves, followed by 0.7mm (IQR = 0.2–1.0) at the 8th month with four test-positive calves. In control calves, median skin thickness change ranged from 0.5mm (IQR = 0.3–0.9) at 4th month to 3.47mm (IQR = 1.4–7.2) at 12th month exposure, with one and 14 test-positive calves, respectively (Figure 5B). There was a significant increase of skin reaction to the DIVA cocktail proteins in both control and vaccinated calves [*F*_(1.7, 64.3)_; *p* < 0.001] during the exposure period though the skin reaction was stronger in control calves [*F*_(1, 38)_; *p* < 0.01]. However, similar to ESAT-6/CFP-10-based IGRA response, the difference between vaccinated and non-vaccinated calves at each post-exposure testing point was not found to be statistically significant after correcting for multiple comparisons (Bonferroni test, *p* > 0.05).

#### Humoral Immune Response

**Figure 6:**
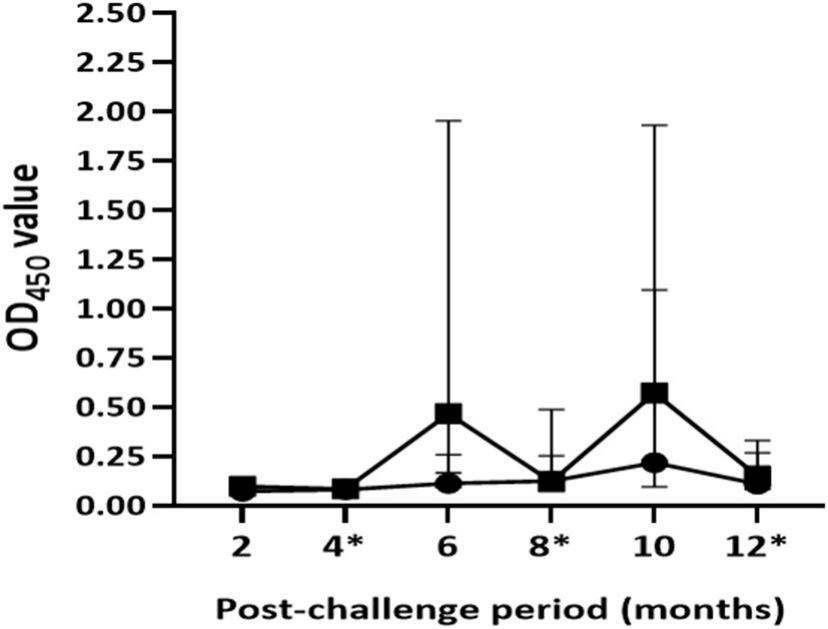
Post-exposure antibody responses in the experimental calves. The figure shows dynamics of the humoral immune response in the vaccinated and control calves during 12 months of exposure. Results were expressed by OD_450_ value of the antibody response. Solid circle and solid square error bars indicate the median (±95% CI) OD_450_ value of vaccinated and control calves, respectively. The magnitude of antibody responses significantly (*p* < 0.001) increased in the vaccinated calves at the 6th and the 10th month compared to those of the control calves. Asterisks indicate months at which the skin testing was done after exposure.

Antibody responses were undetectable in both groups of calves after 2 months of exposure to infected animals. Median OD_450_ values of antibody response in unvaccinated calves ranged from 0.07 (IQR = 0.06–0.08) at the 2nd month to 0.22 (IQR = 0.10–1.20) at the 10th month post-exposure (Figure 6). In this group, antibody responses started to appear at the 4th month with two test-positive calves; then the number of positive calves was seven, six, five, and four at the 6th, 8th, 10th, and 12th month, respectively. In contrast, positive antibody responses were not observed in the vaccinated calves until 6 months of exposure. The magnitude of antibody responses and number of test-positive calves significantly (*p* < 0.001) increased in vaccinated group at the 6th and 10th month compared with that of control calves. The median OD_450_ value response drastically increased to 0.47 (IQR = 0.16–2.04) at the 6th month with 12 positive calves and then decreased to 0.12 (IQR = 0.09–0.50) at month 8 with seven positive calves, after which it rose to 0.57 (IQR = 0.20–1.94) at the 10th month with 12 positive calves (Figure 6). At the end of the experiment, median OD_450_ values reduced to 0.15 (IQR = 0.08–0.37) with only five test-positive calves. Furthermore, there was no correlation (*r* =0.093, *p* > 0.05) between antibody response at the end of the experiment and pathological score.

### Efficacy of BCG Vaccine in Protecting Against Bovine Tuberculosis

#### Pathological Examination

**Figure 7:**
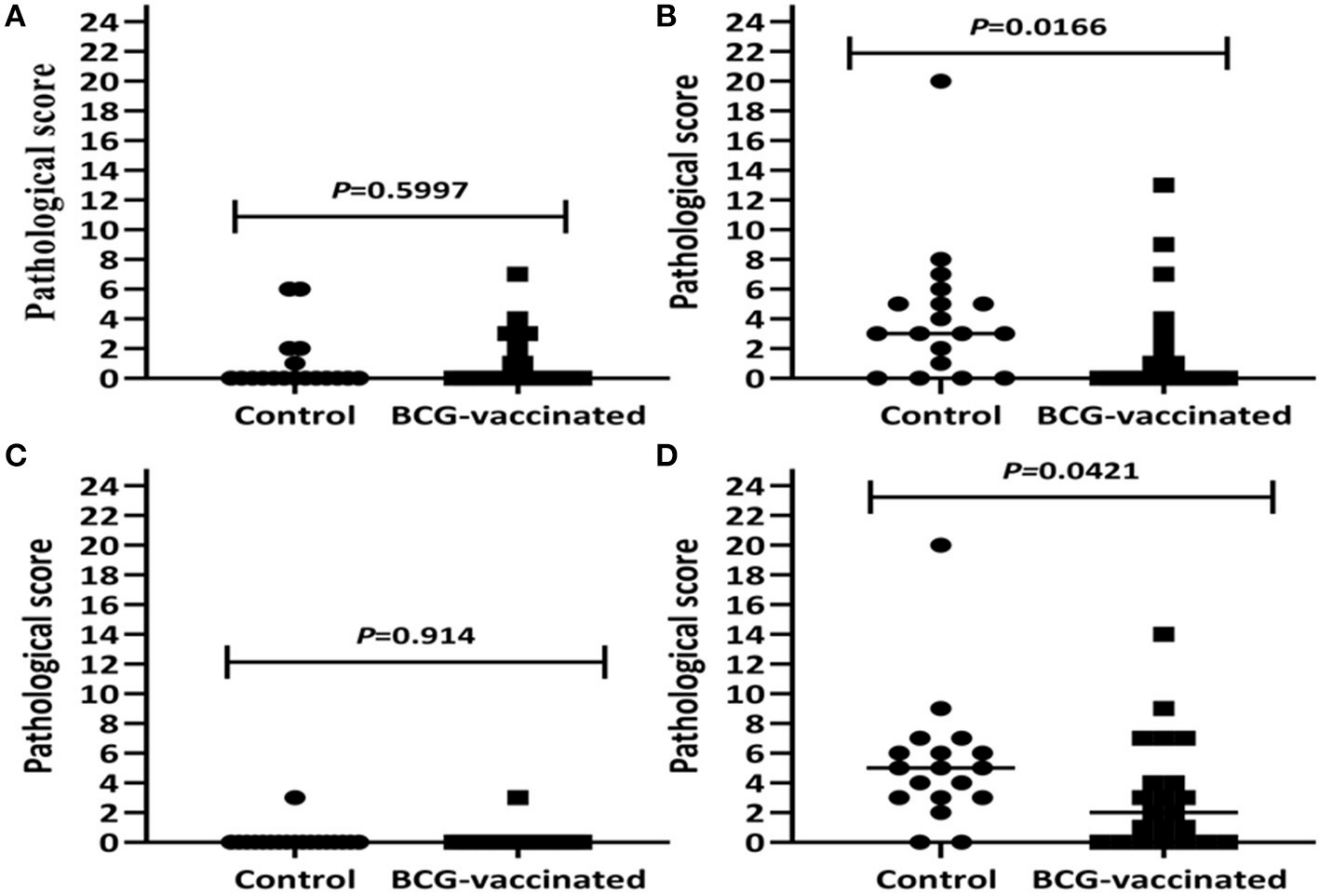
Pathology scores of the experimental calves. Comparison of pathology scores of lymph nodes of the head region **(A)**, the thoracic lymph nodes and lungs **(B)**, abdominal lymph nodes **(C)**, as well as the total pathology score **(D)** in the vaccinated and control calves are presented by the figure. The individual score is represented by a solid circle for the control group and a solid square for the vaccinated group, and medians are indicated by horizontal line. The total pathology score was significantly (*p* < 0.05) lower in the vaccinated calves than in the control calves that was due to the reduction of the pathology score in the tissues of the thoracic cavity.

As presented in Table 1, visible TB lesions were found in 89% (95% CI = 65–99) of the control calves and 68% (95% CI = 45.1–86.1) of the vaccinated calves. Thus, the direct protective efficacy of BCG was 23% (95% CI: −7.5 to 45.3) based on visible TB lesions. There was higher total median pathology score in the control than in the vaccinated group, and the difference was statistically significant (*p* < 0.05) (Figure 7D). The total sum of the pathology score in the control calves was 95 (median 5, IQR = 3.0–6.3) and 68 in the vaccinated calves (median 2.0, IQR = 0–4.8), indicating that vaccination reduced the overall pathology score by 38% (95% CI = 5.4–59.6) (Table 2). This significant reduction of the pathology score was observed in the tissues of the thoracic cavity only (Figure 7B) while there were no significant differences in pathology scores of the head and the abdominal lymph nodes of the vaccinated and control groups (Figures 7A,C). Likewise, the percentage of lesioned tissue was 7.0% (34/484) in vaccinated calves and 11.4% (45/396) in control calves with a significant (38.2%, 95% CI = 5.8–59.4, *p* < 0.05) reduction in the frequency of lesioned tissue in the vaccinated calves (Table 2). Such drop was due to the reduction (54.2%, 95% CI = 23.0–72.8) in the thoracic cavity tissues.

**Table 1:** Protective efficacy of the BCG against bTB in HF × Zebu crossbred BCG-vaccinated and control calves after 1 year of exposure to SICCT test positive cows.

**Parameter**	**No. (%) of control calves (*n* = 18)**	**No. (%) of vaccinated calves (*n* = 22)**	**Protective efficacy**	
			**%**	**95% CI**
Visible TB lesion	16 (88.9)	15 (68.2)	23.3	−7.5–45.3
Histopathology	16 (88.9)	13 (59.1)	33.5	2.2–54.7
Presence of AFB in infected tissue	12 (66.7)	7 (31.8)	52.3	4.5–76.1
*M. bovis* culture positivity	16 (88.9)	14 (63.6)	28.4	−2.7–50.1
ESAT-6/CFP-10 based IGRA^*^	16 (72.7)	17 (94.4)	23.0	−1.8–41.8

* *IGRA after 12 months exposure*.

**Table 2:** The percentage of TB macroscopic lesions in BCG vaccinated and control calves after exposure to an infected herd in a natural transmission setting.

**Tissue**	**BCG-vaccinates proportion of lesioned tissues**	**Controls proportion of lesioned tissues**	**Reduction in frequency of pathology**	**Chi-square test**	***p-*value**
Head lymph nodes (LNs)	7.95% (14/176)	7.64% (11/144)	−4.13%	0.011	0.9166
Thoracic LNs	11.82% (13/110)	24.44% (22/90)	51.65%	5.466	0.0194^*^
Abdominal LNs	2.27% (1/44)	2.78% (1/36)	18.18%	-	>0.999
Lung lobes	3.90% (6/154)	8.73% (11/126)	55.37%	2.840	0.0920
Total pathology	7.02% (34/484)	11.36% (45/396)	38.18%	5.018	0.0251^*^

* *p < 0.05*.

#### Histopathological Examination

**Figure 8:**
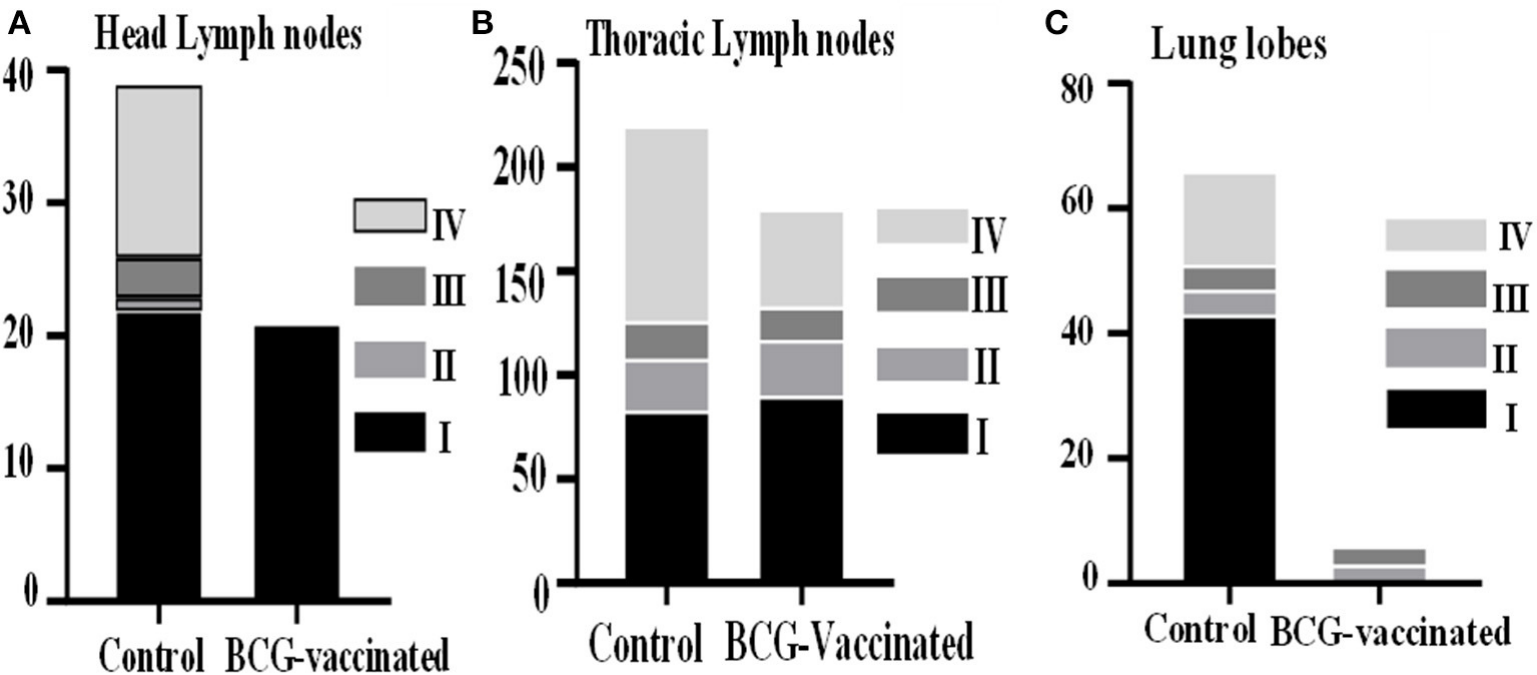
Microscopic lesion scores of the experimental calves. The Figure shows the frequency of occurrence of the different granuloma stages (I–IV) within the head lymph nodes **(A)**, the thoracic lymph nodes **(B)**, and lung lobes **(C)** of control and vaccinated calves. The bars represent the percentage of granulomas with different stages.

Microscopically, 89% (95% CI = 65–99) of the non-vaccinated calves had different stages of granulomas in infected tissues while the granulomas were seen in 59.1% (95% CI = 36.4–79.3) of the vaccinated cattle. Thus, the vaccine protected 33.5% (95% CI = 2.2–54.7) of the vaccinated calves from developing TB granulomas. The frequency of occurrence of granuloma stages in affected head and thoracic lymph nodes, and lung lobes are presented in Figure 8. A total of 558 individual granulomas (226 from 16 vaccinates and 332 from 16 controls) were detected. Except the 26 granulomas from mesenteric lymph nodes, the majority of granulomas were found in the head lymph nodes, thoracic lymph nodes, and lung lobes. Overall, 51.3% (*n* = 116) of the granulomas found in affected tissues of the vaccinated calves were at stage I, while the corresponding stage I in the control calves was 46.1% (*n* = 153). Thus, the frequency of stage I granuloma was significantly (*p* < 0.01) higher in the vaccinated calves than that of the control calves. On the other hand, a significantly higher stage IV granuloma (*n* = 112, *p* < 0.01) was recorded in affected tissues of the control calves as compared with its occurrence in the vaccinated calves (*n* = 60). On the other hand, stages II and III granulomas were comparable in affected tissues of both groups of calves.

Acid-fast bacilli were detected within affected tissues of seven vaccinated calves (31.8%, 95% CI = 13.9–54.9) and 12 non-vaccinated calves (66.7%, 95% CI = 41.0–86.7), resulting in efficacy of 52.3% (4.5–76.1). However, the difference in the prevalence of AFB in the affected tissue between the groups was not significant. The median count of AFB in the granuloma of the control calves was 3.0 while it was 1.4 in the granuloma of the vaccinated calves; the difference between the two was significant (Mann–Whitney *U*-test, *p* < 0.05).

#### Bacteriological Examination

A total of 67 isolates were obtained from culturing of lymph node and lung tissues collected from 34 calves. Out of these, 54 isolates that were recovered from 30 calves were *M. bovis* based on RD4 deletion typing and the dominant spoligotypes were B0912 and SB0913. The remaining isolates could not give interpretable spoligotype patterns. Group-wise, *M. bovis* was isolated from 88.9% (95% CI = 65.3–98.6) of the control calves while it was isolated from 63.6% (95% CI: 40.7–82.8) of the vaccinated calves (Table 1). Thus, the direct protective efficacy of BCG was 28% (95% CI = −2.8 to 50.1) based on *M. bovis* culture positivity. Nonetheless, the difference in prevalence of *M. bovis* culture positivity was not significant (Fisher's exact-test*, p* > 0.05) between the two groups.

#### Survival From M. Bovis Infection

**Figure 9:**
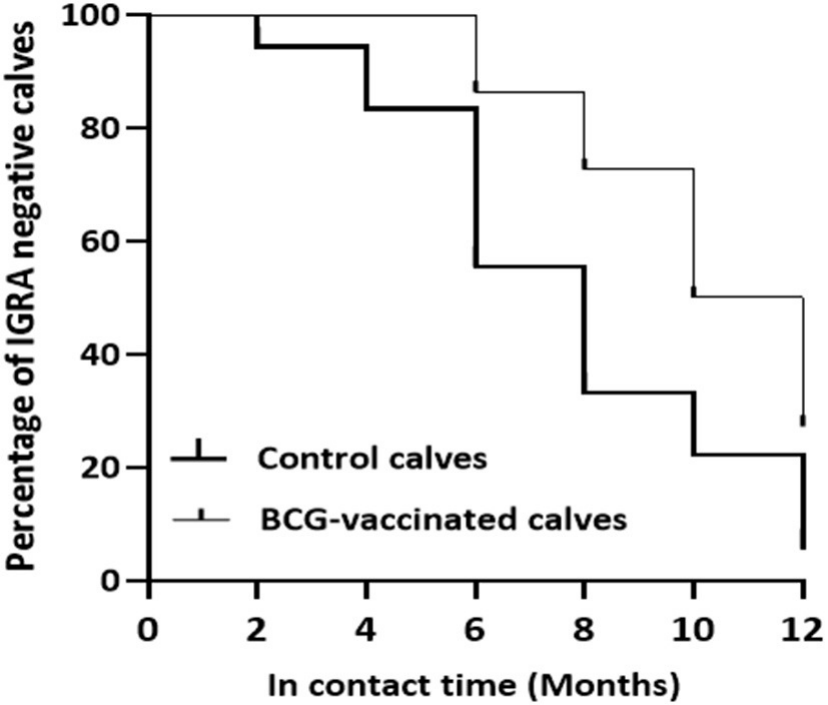
Survival of the experimental calves from *Mycobacterium bovis* infection. The figure shows reduction in the percentages of specific peptides cocktail-based IGRA negative calves in the vaccinated and control groups during exposure. The thick line curve represents the control calves while the thin line curve for the control calves. There was a significant difference (Log-rank test: χ^2^ = 6.749, *P* < 0.01) in the rate of survival from *M. bovis* infection between the vaccinated and control calves.

Based on the specific peptides cocktail-based IGRA result, the prevalence of *M. bovis* infection was 94.4% (95% CI = 72.7–99.9) in the control while it was 72.7% (95% CI = 49.8–89.3) in the vaccinated calves, resulting in direct efficacy of 23.0% (95% CI = −1.8 to 41.8) (Table 1). The survival analysis estimated that median duration surviving from *M. bovis* infection was 8 months of exposure to bTB-infected herd for non-vaccinated calves and 11 months from BCG-vaccinated calves. There was also a significant difference [log-rank test: χ${}_{\left(1\right)}^{2}$ = 6.749, *p* < 0.01] in the rate of staying negative in the IGRA test between control And vaccinated calves (Figure 9).

## Discussion

In the present study, the efficacy of the BCG in protecting against bTB was evaluated in Ethiopian HF × zebu calves under a natural contact challenge setting. The protective efficacy of BCG was estimated using specific peptide cocktail–based IGRA, gross pathology, histopathology, *M. bovis* culture positivity, and survival from *M. bovis* infection. The neonates were vaccinated with the Russian strain of BCG at 2 weeks of age to minimize the risk of interference of environmental mycobacterial infection with the BCG vaccination. The absence of detectable response to the ESAT-6/CFP-10 peptide cocktail in the IFN-γ assay and to the DIVA skin test confirmed that all experimental calves were free from *M. bovis* infection before the start of the experiment. On top of this, post-vaccination SICCT test positivity in the vaccinated neonates indicated the successful uptake of the vaccine.

The evaluation of the efficacy of BCG was conducted by housing the vaccinated and control calves for 1 year with cows that were all SICCT test positive. After exposure to the infected cows for 1 year, the efficacy ranging from 23.3% based on visible lesion to 52.3% on the basis of the detection of AFB in the affected tissues was recorded. Similarly, the observed efficacy on the basis of *M. bovis* positivity was 28.4%. Substantiating the finding of this study, an efficacy of 28% was reported in HF calves in Ethiopia a few years ago (12). However, the current recorded efficacy was considerably lower than the efficacy reported by other similar studies from Ethiopia (5), Mexico (24), New Zealand (38), and Chile (39). The difference in the BCG efficacy recorded by different studies can be due to variation in setting of the experiment, strains of BCG used for vaccination, severity of disease in the seeder animals, the sample size of experimental calves, the pressure of transmission (ratio of calves to seeders), age of calves at vaccination, breeds of the calves, the extent of detailed post-mortem examination performed, the magnitude contacts maintained between the calves and the seeders, etc. For example, regarding the two previous Ethiopian studies, the first study (5) was conducted on relatively few numbers of calves and a higher efficacy was recorded. However, the sample size of the second study (12) and that of this present study were relatively larger and similar.

Similarly, although the seeder cows were recruited based on results of the SICCT test, this test and other ante-mortem bTB diagnostic tests cannot definitively indicate the disease status of the seeders (17, 40), which makes it difficult to estimate the pressure of infection created by seeder cows used in different studies. Moreover, cattle-to-cattle transmission of *M. bovis* requires close contact of the susceptible animal with sources of infection (41). Hence, the higher efficacy of 59% recorded in the Mexican study may be related to the use of a reactor herd with 40% prevalence for the challenge (24), which could have caused a less transmission pressure as compared with the studies that have used 100% reactor herds, as it was the case in the present study. Furthermore, the 67% efficacy estimation in a large-scale field trial in New Zealand was likely related to the setting of the study, in which the seeders and the naive animals were grazing on pasture (38), in which the infection pressure was obviously low. In addition, a recent study in Chile under a routine farm practice reported a BCG efficacy of 66.5% (39) on the basis of specific *M. bovis* antigen-based IGRA in the field condition. Similar to the study in New Zealand, the infection pressure was low as contact challenge was conducted in a herd with bTB prevalence of 24%. Although the efficacy value recorded by the present study was within the range of BCG efficacy (0 and 75%) reported from experimental studies and field trials in cattle by different researchers from different countries [reviewed by (19, 21)], the use of 100% positive seeders as a source of infection could have contributed to the lower efficacy value recorded, as compared with those studies conducted in Mexico, New Zealand, and Chile.

It is well-established that BCG vaccination does not confer absolute protection against *M*. *bovis* infection in cattle [reviewed by (20, 21)]. Instead, it can considerably reduce the development of lesions and its progression in infected tissues (5, 12). A similar observation was made in the present trial, which evaluated the efficacy of BCG in HF × zebu crossbred cattle for the first time. Thus, the gross and histopathological examinations revealed a significant reduction of the number of infected tissues and/or severity of lesions in the vaccinated calves. Besides, the count of AFB was significantly decreased in the affected tissues of vaccinated calves as compared with those of the control calves. In support of the present finding, a study conducted in Spain reported that the proportion of AFB positivity in mesenteric lymph nodes was significantly lower in BCG-vaccinated goats than in the control goats (42). Such findings suggest that BCG may play a role in limiting the multiplication of mycobacteria within infected tissues. On top of these, BCG vaccination could improve the incidence rate *M. bovis* infection in the vaccinated calves than that of the control calves. A similar effect of BCG was observed in a recent field study conducted in Chile (39). Hence, BCG vaccination could play an important role in containing the onward transmission of bTB from vaccinated cattle to susceptible cattle.

The kinetics of the cell-mediated immune responses after BCG vaccination and *M. bovis* infection was studied using the SICCT test and the IGRA. After vaccination, IFN-γ responses to PPD (B-A) reached a peak at the third week and remained at higher level until the sixth week post-vaccination. However, no detectable IFN-γ response to the specific ESAT-6/CFP-10 peptide cocktail was observed in vaccinated calves during post-vaccination, which can demonstrate the DIVA role of the ESAT-6/CFP-10 peptide cocktail while PPD could not achieve this role. Earlier studies conducted in Ethiopia also reported PPD-B biased strong IFN-γ response post-BCG vaccination (43, 44) although non-specific IFN-γ response in calves at early age (45) and exposure to environmental mycobacterial antigens (18) could affect the IFN-γ response to PPD-B.

After exposure to infected cows, IFN-γ response to the specific peptide cocktail was monitored every 2 months until the end of experiment. During this period, a progressive increase in the IFN-γ response was observed in both control and vaccinated calves although the response was stronger in the control calves. After the completion of the exposure, calves with stronger IFN-γ response were observed to have more severe pathology as evidenced from positive moderate correlation recorded between IFN-γ response and pathology score. Therefore, it can be suggested that the intensity of the specific peptide cocktail–based IFN-γ responses may not be an indicator of protection associated with BCG vaccination. Similarly, other studies reported that the intensity of IFN-γ responses, after BCG vaccination, did not correlate with protection in cattle (46, 47), suggesting that IFN-γ response alone is not sufficient for protection. One of the possible reasons for such absence of correlation could be associated with duration of immunity (DoI) after BCG vaccination. In this regard, the DoI after vaccination could be shorter and thus, the immunity wanes while the animals are still under exposure to infection. These studies reported variable DoI after BCG vaccination; one of the studies suggested 6 months (30) while the other reported 12 months (48) of DoI. In the present study, most of the vaccinated calves generated strong IFN-γ responses to ESAT-6-/CFP-10-specific antigens after about 6 months of exposure. In agreement with this observation, BCG-vaccinated goats became infected in less than a year of exposure to infected goats in Spain (49). Thus, these observations could suggest that the DoI of BCG is <1 year requiring a boosting dose.

On the other hand, defined proteins skin test demonstrated its suitability as a DIVA skin test as none of vaccinated calves were positive at the 6th week of vaccination. However, the diagnostic sensitivity of DIVA skin test was low, especially in vaccinated calves, compared with the SICCT test. The reason for low sensitivity of the DIVA skin test is unclear but could be associated with the antigenicity of the DIVA proteins used, repeated testing, and/or immunological shift from cellular to humoral response.

The role of the humoral immune response against bTB and as the parameter for vaccine efficacy evaluation has been understudied (49). However, B cells are observed in cattle infected with *M. bovis*, suggesting that antibodies may play a significant role in the control of mycobacterial infection (50). In the present experiment, eight calves (36.4%) generated an antibody response to BCG vaccination at the 6th week of vaccination. Similar observations were made by previous studies (45, 50–52). The widely used proteins for serological tests are MPB70 and MPB83 that are expressed in low level in the BCG strains (53, 54) as compared with their expressions in the virulent *M. bovis* strains, suggesting their roles as DIVA reagents (55). However, in the present study, eight vaccinated calves before exposure to the infected cows tested positive in the IDEXX *M. bovis* ELISA based on these proteins, which could undermine the claim of DIVA role of these proteins.

The first antibody reactive calves were detected at the 4th and the 6th months post-exposure in the control and vaccinated calves, respectively. At the end of the experiment, out of 16 control calves with visible lesion, only five were positive by the IDEXX *M. bovis* ELISA. Similarly, eight calves with visible lesion from 15 vaccinated calves were not positive with IDEXX *M. bovis* ELISA. Hence, these figures suggested lower diagnostic sensitivity of the IDEXX *M. bovis* ELISA for detection of *M. bovis* infection. In agreement with the findings of the present study, previous studies indicated that serological tests including the IDEXX *M. bovis* ELISA have low sensitivity when they are done before a tuberculin skin test (53, 56). In the present study, the level of antibody response and the proportion of animals positive for serology in vaccinates significantly increased after 2 months of tuberculin and DIVA skin tests. Similar to this observation, a recent study indicated a significant increase in seropositivity in calves vaccinated with killed or live BCG 1 month after tuberculin skin test (50). Although previous observation indicated a positive correlation between the antibody response to *M. bovis* infection and severity of the TB disease (57, 58), such observation was not demonstrated by the present study.

## Conclusion

In conclusion, although the direct protective efficacy of the BCG in protecting against bTB in HF × Zebu crossbreed calves was low, it was demonstrated that BCG vaccination reduced the severity and dissemination of lesions, and also delayed *M. bovis* infection in the vaccinated calves. These impacts of BCG vaccination could contribute to the containment of onward transmission of *M. bovis* from vaccinated animals to other susceptible animals.

## Data Availability Statement

The raw data supporting the conclusions of this article will be made available by the authors, without undue reservation.

## Ethics Statement

The animal study was reviewed and approved by Institutional Review Board, Aklilu Lemma Institute of Pathobiology, Addis Ababa University, Ethiopia.

## Author Contributions

BB and AS collected and analyzed the data and drafted the article. GA, HV, RH, AC, SB, and JW conceived and designed the study and edited the article. AW, AZ, YZ, and MC assisted in the field and laboratory data collection. AC, HV, and GA contributed in the analysis of the data. BG edited the article. All authors reviewed the final draft and agreed on its content and conclusions.

## Conflict of Interest

The authors declare that the research was conducted in the absence of any commercial or financial relationships that could be construed as a potential conflict of interest.

## Publisher's Note

All claims expressed in this article are solely those of the authors and do not necessarily represent those of their affiliated organizations, or those of the publisher, the editors and the reviewers. Any product that may be evaluated in this article, or claim that may be made by its manufacturer, is not guaranteed or endorsed by the publisher.
